# iPS-derived MSCs from an expandable bank to deliver a prodrug-converting enzyme that limits growth and metastases of human breast cancers

**DOI:** 10.1038/cddiscovery.2016.64

**Published:** 2017-02-06

**Authors:** M Ullah, Y Kuroda, T J Bartosh, F Liu, Q Zhao, C Gregory, R Reger, J Xu, R H Lee, D J Prockop

**Affiliations:** 1 Institute for Regenerative Medicine at Scott & White, Texas A&M University and Health Science Center, College of Medicine, Temple, TX 76502, USA

## Abstract

One attractive strategy to treat cancers is to deliver an exogenous enzyme that will convert a non-toxic compound to a highly toxic derivative. The strategy was tested with viral vectors but was disappointing because the efficiency of transduction into tumor cells was too low. Recent reports demonstrated that the limitation can be addressed by using tissue-derived mesenchymal stromal cells (MSCs) to deliver enzyme/prodrug systems that kill adjacent cancer cells through bystander effects. Here we addressed the limitation that tissue-derived MSCs vary in their properties and are difficult to generate in the large numbers needed for clinical applications. We prepared a Feeder Stock of MSCs from induced pluripotent stem cells (iPSs) that provided an extensively expandable source of standardized cells. We then transduced the iPS-derived MSCs to express cytosine deaminase and injected them locally into a mouse xenogeneic model of human breast cancer. After administration of the prodrug (5-fluorocytosine), the transduced iPS-MSCs both limited growth of preformed tumors and decreased lung metastases.

## Introduction

Among the many strategies suggested for the therapy of cancers is the use of an exogenous enzyme to convert a non-toxic compound to a highly toxic derivative, a strategy referred to as gene-directed enzyme/prodrug therapy (GDEPT).^1,2^ The strategy was initially introduced with the advent of viral gene therapy in the 1990s and extensively tested over >20 years. However, clinical trials using viral vectors were disappointing. The major limitation was the low efficiency of transduction of tumor cells. Solid tumors were particularly resistant because, even with local injection, the viruses did not transduce many cells beyond the needle track. Subsequently, interest in the GDEPT strategy was revived with the possibility of using cells to deliver either viruses with the critical gene or the prodrug-converting enzyme itself.^3^ Among the cells that have been most extensively tested are the progenitor cells referred to as mesenchymal stem/stromal cells (MSCs).

MSCs have several attractive features as vectors of GDEPT.^3–5^ Human MSCs can be readily obtained from bone marrow, fat, umbilical cord and synovial membranes; they can be expanded in culture; they can be genetically transduced; and they are relatively non-immunogenic.^6,7^ Unlike induced pluripotent stem cells (iPSs), they senesce in culture and therefore pose a minimal risk of being tumorigenic. Also, part of the appeal for GDEPT is that they tend to home to tumors and become part of the tumor environment as tumor-infiltrating cells.^8,9^ However, MSCs enhance tumor growth and metastases under some experimental conditions.^10–12^ In spite of this concern, essentially no adverse events have been reported with administration of MSCs that met standard criteria to large numbers of patients in >200 clinical trials.^13^

Because of these features, MSCs transduced to express suicide or killer genes with bystander effects were used in a series of xenogeneic experiments with cancer cells.^4,5^ Most of the reports have used MSCs that express thymidine kinase from herpes simplex virus that initiates conversion of ganciclovir or related substrates to phosphorylated derivatives that inhibit DNA synthesis (TK/GCV) or cytosine deaminase (CD) from yeast that converts 5-fluorocytosine to chemotherapeutic agent 5-fluorouracil that is further metabolized within cells and incorporated into DNA and RNA (CD/5-fluorocystine (5-FC)). Of the two, the CD/5-FC system had the advantage that 5-fluorouracil freely diffuses from the transduced cells, whereas phosphorylated ganciclovir metabolites from TK/GCV require gap junctions for transmission to cancer cells,^14,15^ but combinations of the two systems were more effective for inhibition of resistant cancers in some experimental situations.^16^ Also, most of the experiments were performed with transduced MSCs co-injected with cancer cells or injected directly into or near preformed tumors. Inhibition of tumor growth was consistently observed. In one report, long-term efficacy in suppressing a melanoma was demonstrated by combing MSCs transduced to express CD with an inhibitor of cMet/hepatocyte growth factor signaling.^17^ In effect, the results demonstrated that local injection of transduced MSCs overcame the problem of delivering adequate amounts of the prodrug-converting enzyme to tumors. Intravenous (IV) administration was also effective in several reports. Lung metastases produced by IV administration of cancer cells were reduced by IV administration of GDEPT MSCs,^16,18^ an observation consistent with the evidence that most MSCs are trapped in the lung after IV infusion.^19^ IV infusions of transduced MSCs were also observed to inhibit growth of cancers injected subcutaneously.^20,21^ However, the homing of MSCs to cancers is not highly efficient,^19^ and the IV route raises the possibility that it will lower the therapeutic window for the prodrug because the transduced MSCs trapped in other tissues will generate high local levels of the toxic drug and produce unwanted effects on normal cells.

In effect, multiple reports have established the efficacy of local injections of MSCs as vectors for GDEPT. However, a number of issues will need to be addressed in translating the results obtained in mouse xenogeneic models to patients. Two critical issues are the variability encountered with the tissue-derived MSCs and the need to generate the very large number of MSCs required for many clinical applications.

The variability of tissue-derived MSCs is loosely referred to as ‘donor variability’,^22–25^ but it is observed in bone marrow aspirates obtained from the same donor at the same setting^6^ and different fat deposits.^25^ Therefore, part of the variation is a result of unguided sampling of tissues. The variability of the MSCs is not only apparent in their varying yields from tissues but also in *in vitro* properties that include their rates of proliferation, clonogenicity, potential for differentiation and the extent to which the MSCs can be expanded in culture. The variability is also apparent in the few experiments in which the efficacies of different preparations have been compared quantitatively in animal models.^24^ Use of biomarkers such as expression of the inflammation-modulating protein TSG-6 may help select optimally effective preparations for some disease targets,^24^ but the development of biomarkers for MSCs is still in its infancy.

One strategy for overcoming the variability of tissue-derived MSCs is to prepare large banks of MSCs from a single donor. The size of the banks, however, is limited by several biological constraints. High initial yields of MSCs can be obtained from tissues, such as fat, umbilical cord and large bone marrow aspirates. The cells can be further expanded in culture within a biological window that varies among different preparations and with culture conditions. For example, optimal preparations of MSCs from human bone marrow^26,27^ expand rapidly through about 30-population doublings (2^30^=1 billion fold expansion). Thereafter, their rate of propagation slows and they approach cell cycle arrest in a senescent-like state. Use of MSCs for GDEPT, however, requires both genetically transducing the cells and extensively expanding them after transduction. The combined manipulations of transduction and expansion introduce new sources of variability, such as changes in the properties of the MSCs,^23,28^ different levels of expression of the transduced gene,^15^ possible shut-down of the transduced gene or selection for non-transduced cells with expansion, site-directed mutagenesis and alterations that change the survival of the cells *in vivo*. Such variations pose special problems for use of MSCs in GDEPT, as the transduced MSCs must be titered to both the number of MSCs engrafted and the administered dose of the prodrug *in vivo*. A number of efforts are still in progress, particularly by start-up biotechnology companies, to develop therapies with large banks of tissue-derived MSCs from single donors, but it is not apparent whether the biological window for the expansion of MSCs may limit their size and therefore their applications to common diseases.

Another strategy to address the variability of tissue-derived MSCs and the need to generate large numbers of the cells is to begin with iPSs and differentiate them to MSCs.^29–35^ The strategy offers the prospect of generating an essentially unlimited number of cells because the iPS cells can be expanded without limit before differentiation to MSCs. Also, if desirable, it offers the prospect of inserting genes for GDEPT or for other therapies into safe harbors with CRISPR/Cas9 and related technologies^36,37^ that minimize insertional mutagenesis and shut-down of expression as the cells are expanded. In the experiments described here, we tested the hypothesis that iPS-derived MSCs from a Feeder Stock that was prepared with the modified protocol^19^ can serve as a renewable source of standardized cells for therapy of breast cancers with GDEPT. We transduced the cells with a lentiviral vector to express CD. We then found that local injection of the transduced cells together with systemic administration of the prodrug not only limited growth of the cancer but also decreased lung metastases in a xenogeneic model for breast cancer. The injected iPS-derived MSCs rapidly disappeared from the tumors even without administration of the prodrug, an observation that confirmed previous indications that the cells are unlikely to produce adverse events in patients.^9^

## Results

### Preparation and transduction of iPS-derived MSCs

**Figure 1:**
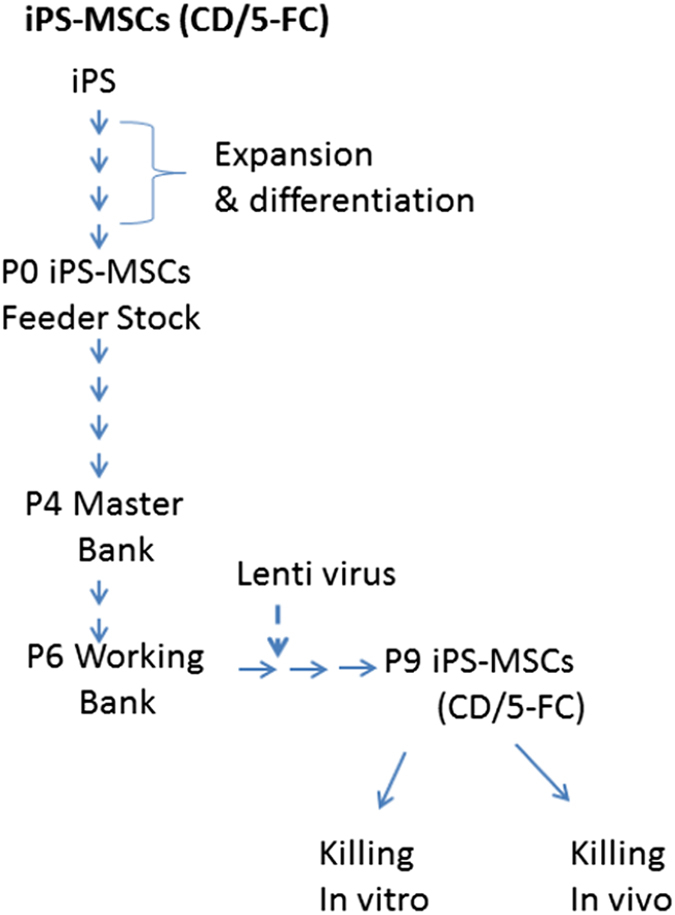
Schematic representation of the Master Bank cells. iPS-derived MSCs were expanded from the P0 Feeder Bank to generate cells for the P6 Working Bank. Cells from the P6 Working Bank were expanded to P7 cells and then P7 cells were transfected with a lentivirus designed to express genes for CD gene and UPRT (CD::UPRT). As indicated in the text, cells from the P0 Feeder Bank could be expanded about 10^6^-fold to generate cells for the P6 Working Bank as needed.

As recently described,^9^ iPS-derived MSCs were prepared from iPS cells generated from whole blood with episomal vectors. The cells were expanded and differentiated through four steps to obtain iPS-MSCs as defined by the loss of expression of embryonic genes and acquisition of features characteristic of MSCs, including spindle-like morphology, typical surface epitopes, clonogenicity and differentiation to osteoblasts and chondrocytes in culture. The first cultures that satisfied these criteria were designated as passage 0 (P0) iPS-MSCs (Figure 1). P0 iPS-MSCs were stored frozen as a Feeder Bank cells for generation of Master Banks as required.

For the experiments here, the P0 cells from the Feeder Bank were expanded to obtain a P6 Working Bank. For the first five passages, the cells were plated at 500 cells/cm^2^ and incubated for 3–5 days. The P5 cells were expanded once through a perfusion bioreactor (Quantum; Terumo, Lakewood, CO, USA) to provide the P6 Working Bank. The average fold expansion per passage was 9.5±1.80 S.D., and the average population doublings per passage was 3.26±0.317 S.D. Therefore, the P0 cells in the Feeder Bank could be readily expanded about 10^6^-fold to generate cells for the P6 Working Bank.

**Figure 2:**
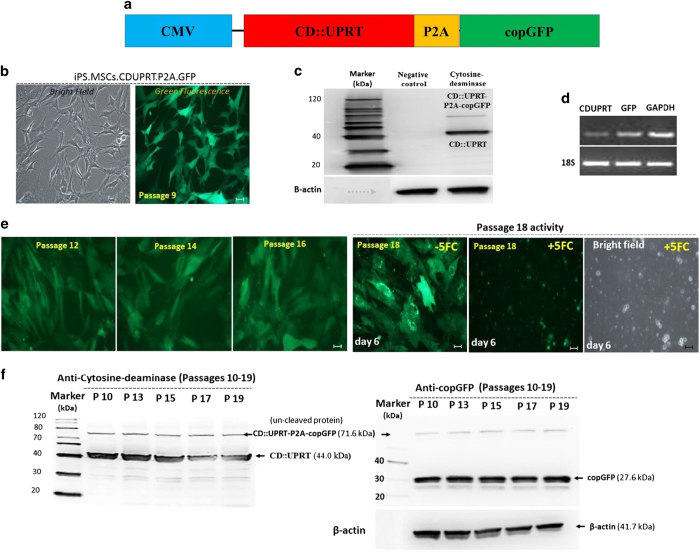
Transduced iPS-MSCs. (**a**) Map of CMV promotor driving expression of the CD::UPRT genes linked with copGFP by P2A sequence for cleavage of the two proteins after translation. (**b**) Representative images of iPS-MSCs-CD cells expanded to passage 9 after transduction at passage 7. (**c**) Western blotting of passage 9 iPS-MSCs-CD cells demonstrated the expression of CD::UPRT together with a small amount of the uncleaved proteins. (**d**) RT-PCR assays confirmed the expression of the transduced genes. (**e**) Representative images of iPS-MSCs-CD cells demonstrating the expression of GFP after expansion to passages 12, 14 and 18. The passage 18 cells were killed by incubation for 6 days in the presence of 300 *μ*M 5-FC. (**f**) Western blotting analyses demonstrated that both the GFP gene and the CD::UPRT genes continued to be expressed as the iPS-MSCs-CD cells were expanded through passage 19. Scale bar: 50 *μ*m.

To transduce the cells, a construct (Figure 2a) was prepared with the CMV promoter driving expression of CD linked to uridine phosphoribosyl transferase (UPRT), a gene combination shown to enhance the expression of CD.^17^ The CD::UPRT fusion protein was followed by P2A, a spontaneous protein cleavage site, and a gene encoding copGFP, a GFP-like protein with enhanced fluorescence and decreased toxicity.^38^ The construct was inserted into a lentiviral vector and the virus used to transduce the iPS-MSCs. Essentially all the cells expressed GFP by UV fluorescence microscopy (Figure 2b). Expression of CD::UPRT was assayed by western blotting (Figure 2c) and the expression of copGFP was verified by RT-PCR (Figure 2d). Of note, there was no evidence of silencing of the exogenous genes as the cells were expanded from passage 9 to passage 19. The cells continued to express GFP as determined by microscopy and both genes as assayed by western blotting analysis (Figures 2c, e and f).

### Efficacy of the transduced cells in killing cells in co-cultures

**Figure 3:**
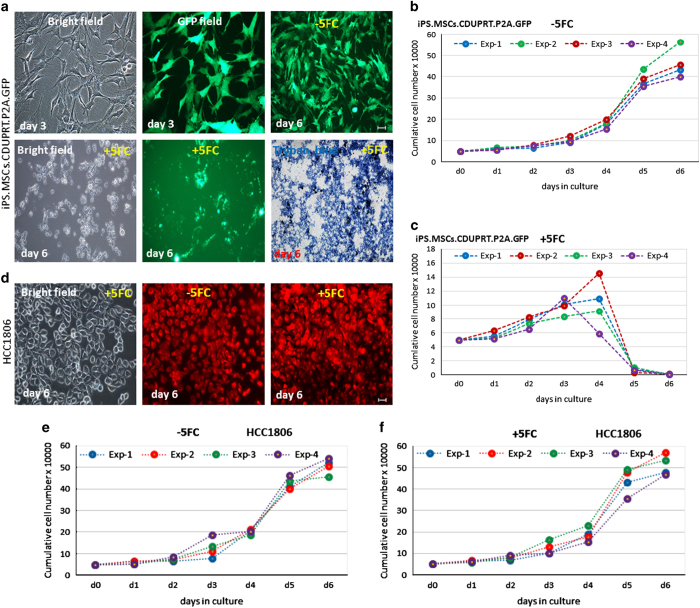
Effects of 5-FC on iPS-MSCs-CDs and HCC1806 in culture. iPS-MSCs-CDs or HCC1806 were cultured for 6 days with or without 300 *μ*M 5-FC. (**a**) Photomicrographs of iPS-MSCs-CDs cultured±5-FC. (**b**) iPS-MSCs-CD cells cultured in the absence of 5-FC. (**c**) iPS-MSCs-CD cells cultured in the presence of 5-FC. (**d**) Photomicrographs of HCC1806 (labeled with CellTracker Red) cultured±5-FC. (**e**) HCC1806 cells cultured in the absence of 5-FC. (**f**) HCC1806 cells cultured in the presence of 5-FC. Scale bar: 50 *μ*m.

**Figure 4:**
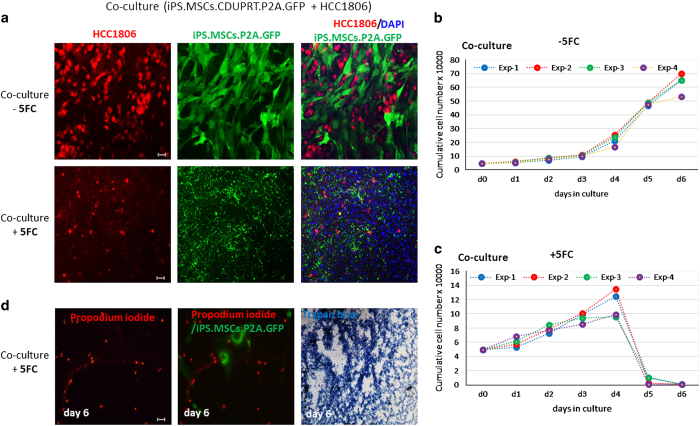
Effects of 5-FC on co-cultures of iPS-MSCs-CD and HCC1806. iPS-MSCs-CD and HCC1806 were co-cultured for 6 days with or without 300 *μ*M 5-FC. (**a**) Representative microscopic images of co-cultures. (**b**) Growth curve of co-cultured cells in the absence of 5-FC. (**c**) Growth curve of co-cultured cells in the presence of 5-FC. (**d**) Photomicrographs of HCC1806 (labeled with propodium iodide) cultured±5-FC. Scale bar: 50 *μ*m.

In initial tests for the efficacy of the transduced cells, we carried out *in vitro* experiments with an aggressive, triple-negative line of breast cancer cells, HCC1806. As expected, the iPS-MSCs-CDs were killed in the presence of 300 *μ*M 5-FC (Figures 3a–c). However, the HCC1806 cells were not (Figures 3d–f). In co-culture experiments, the HCC1806 cells and the transduced cells (iPS-MSCs-CD/5-FC) were plated in a 1 : 1 ratio and then incubated with or without the 300 *μ*M 5-FC. After 6 days, all the cells in the co-culture were killed (Figure 4).

### Efficacy in inhibiting cancer cells after co-injection *in vivo*

**Figure 5:**
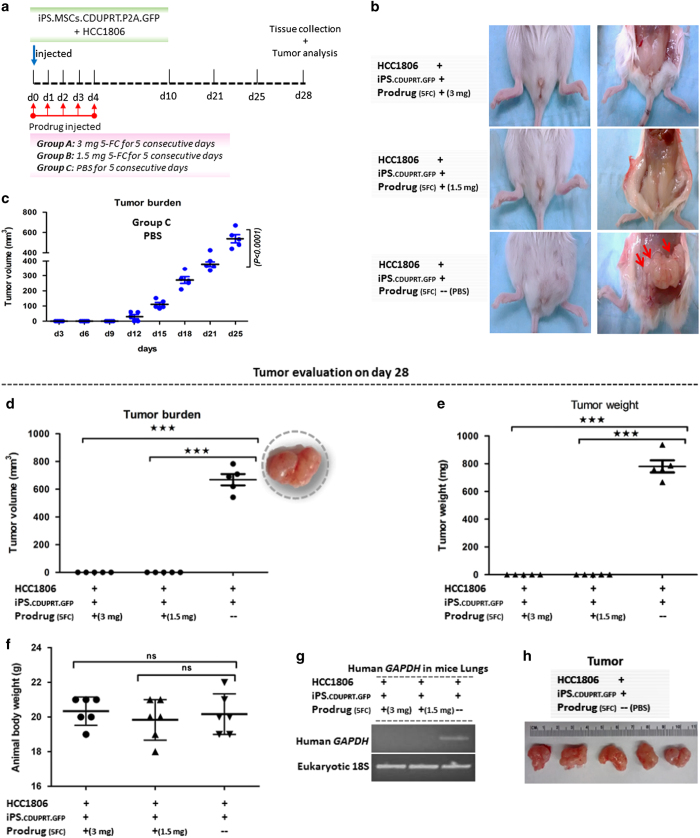
Effects on tumor growth in mice receiving co-injections of iPS-MSCs-CD and HCC1806. A mixture of 1×10^6^ HCC1806 cells and 1×10^6^ iPS-MSCs-CD were injected into a fourth mammary fat pad of each mouse and then 5-FC or PBS was injected i.p. for 5 consecutive days. (**a**) Schematic representation of the experiment. (**b**) Representative images of the mammary glands from treated and control mice before and after killing followed by exposure of the fat pad. (**c**) Growth curve of the tumor burden for 25 days in mice that received PBS instead of 5-FC. (**d**) Tumor volume of the treated and control groups on day 28. (**e**) Tumor weight of the treated and control groups on day 28. (**f**) Body weights of mice from the treated and control groups on day 28. (**g**) RT-PCR assays for human GAPDH signal in the mice lungs of the treated and control groups on day 28. (**h**) Excised tumors of control mice on day 28. Mean (±S.E.M.) tumor growth over time in mice (*n*=5–6 per group) injected with tumor cells and treated as indicated (***P<0.001; NS, not significant).

To test the efficacy of the transduced cells in killing cancers *in vivo*, we injected 1 : 1 mixtures of the HCC1806 cells and the transduced iPS-MSCs-CDs into the mammary fat pads of NOD/*scid* mice (Figure 5a). 5-FC was then injected intraperitoneally (i.p.) daily for 5 days in a dose of either 1.5 or 3 mg per mouse. Control mice were injected with the same mixture of the cells and PBS instead of the prodrug. The control mice developed large tumors (Figures 5b–e) and most had to be killed at 28 days because of deteriorating health. In contrast, mice that were injected with a mixture of the cells together with the prodrug did not develop tumors. None were detected by palpation of the mammary glands during the experiments Figure 5c) and none were detected by dissection of the mammary glands at the end of the experiments (Figures). Similar results were obtained with both doses levels of the prodrug tested. In addition, there was a marked difference in lung metastases. Lung metastases were detected by RT-PCR assays for human glyceraldehyde 3-phosphate dehydrogenase (GAPDH) in five of the five control mice that were injected with the mixture of cells and PBS instead of the prodrug (Figure 5g). In contrast, none were detected with the same assay in 10 mice that received the mixture of cells and either dose of the prodrug (*P*<0.0003, two-tailed Fisher’s Exact test).

### Efficacy in inhibiting growth and metastases of preformed cancers *in vivo*

**Figure 6:**
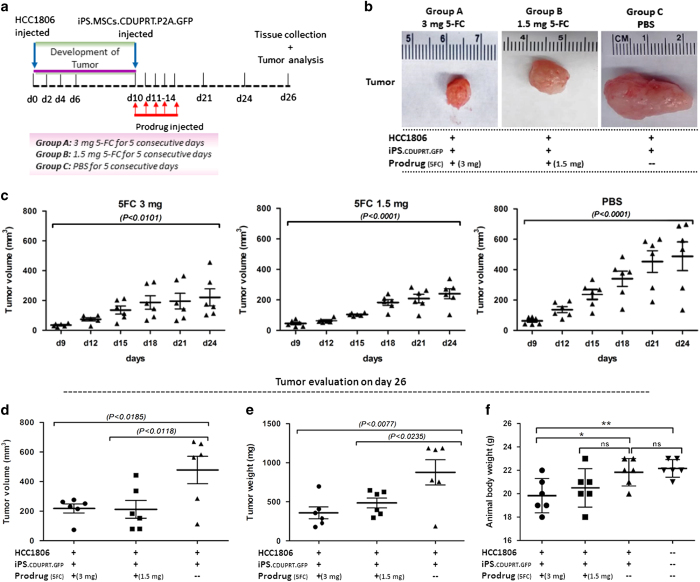
Effects of injecting iPS-MSCs-CD cells into preformed tumors. HCC1806 (1×10^6^) injected into the fat pads of mice and tumors were allowed to grow for 10 days. Then iPS-MSCs-CD (1×10^6^) were injected into the same fat pad or adjacent tissue followed by five daily i.p. injections of 5-FC or PBS. (**a**) Schematic representation of the experiment. (**b**) Representative images of the tumor in the treated and control groups. (**c**) Tumor burden overtime for 24 days in the treated and control groups. (**d**) Tumor volume of the treated and control groups on day 26. (**e**) Tumor weight of the treated and control groups on day 26. (**f**) Body weight of the anaimals of the treated and control groups on day 26. Mean (±S.E.M.) tumor growth over time in mice (*n*=6–7 per group) injected with tumor cells and treated as indicated (*P<0.05; **P<0.01; NS, not significant).

**Figure 7:**
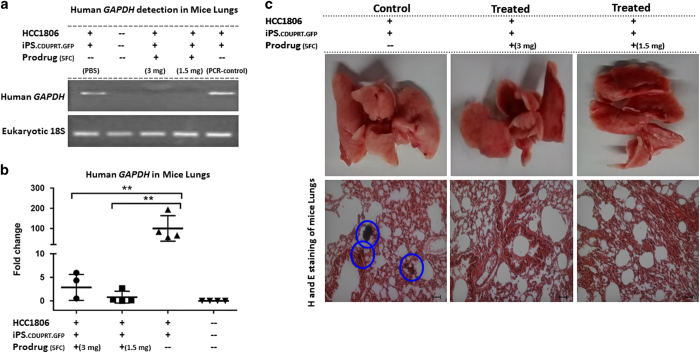
Effects on lung metastases of injecting iPS-MSCs-CD cells into preformed mammary tumors. Assays were performed on the lungs from mice from the experiment described in Figure 6. (**a**) RT-PCR for human GAPDH in the lungs of the treated and control groups on day 26. (**b**) Real time RT-PCR assays of human GAPDH in the lungs of the treated and control groups on day 26. (**c**) Representative images of the whole lung, and H&E-stained sections from the treated and control groups. Tumor nodule-rich areas are encircled (blue) in the control group, while the treated groups shows significant inhibition of lung metastases. Mean (±S.E.M.) tumor growth over time in mice (*n*=6–7 per group) injected with tumor cells and treated as indicated (**P<0.01; NS, not significant), Scale bar: 50 *μ*m.

To test efficacy of the strategy further, we injected HCC1806 cells into the mammary fat pads of the mice and waited for 10 days for the tumors to develop to a size detectable by palpation (Figure 6a). We then injected the transduced iPS-MSCs-CDs into the tumor or adjacent tissue in the same fat pad. I.p. administration of either dose of the prodrug decreased the size of the tumors as detected by palpation during the course of the experiment (Figure 6c). Either dose also decreased the volume and weight of the tumors at the end of the experiment (Figure 6d). Again, there was a marked difference in lung metastases. Assays by RT-PCR for human GAPDH indicated that all four control mice that received PBS instead of the prodrug developed lung metastases (Figure 7). In contrast, the same assays on lungs of seven mice that received either dose of the prodrug did not detect the levels of human GAPDH that differed from the background obtained with control lungs from mice that did not receive any cell injections (Figure 7; *P*<0.003, two-tailed Fisher’s Exact test).

## Discussion

As demonstrated by multiple recent publications,^4,5^ MSCs are attractive vectors for delivering prodrug-converting enzymes to cancers. However, several issues must be addressed before the results can be translated to patient therapies. The data presented here address the critical issue of how to prepare an essentially unlimited number of MSCs with invariant properties that are effective for GDEPT.

iPS-derived MSCs have several features that may make them attractive for some therapeutic applications.^9^ The use of a Feeder Bank as defined here (Figure 1) makes it possible to generate an expandable number of cells that have uniform characteristics. The iPS-derived MSCs were readily transduced with a lentivirus to express levels of CD high enough to produce effective killing of breast cancer cells through a bystander effects both in culture and *in vivo*. Moreover, the transduced genes continued to be expressed through 10 additional passages. Therefore, the protocol, with some minor adjustments, provides a means of providing the large number of transduced cells required for clinical applications.

When co-injected with cancer cells followed by the prodrug, the iPS-MSCs-CD inhibited development of detectable tumors. In addition, the iPS-MSCs-CD were effective even if injected into preformed tumors. The effectiveness was apparent not only by a decrease in tumor size but also by an inhibition of lung metastases. Also of importance was the observation that the injected iPS-MSCs quickly disappeared from the tumors, even without administration of the prodrug. Therefore, the cells are unlikely to cause any adverse events.

The results support previous observations that local injection of the MSCs transduced to express CD followed by 5-FC may be useful as an adjunct therapy for some patients with cancer.^4,5^ In the case of breast cancers, the strategy may be appropriate for two subpopulations of patients who do not elect surgery or other inventions: those with very early growths and those with advanced metastatic disease. If desirable, the strategy pursued here could be expanded to introduce the CD gene into safe harbors in the genome with CRISPR/Cas9 or similar strategies^36,37^ to minimize the risk of insertional mutagenesis and to reduce further the possibility of gene silencing as the cells are expanded.

## Materials and Methods

### Ethics statement

All animal procedures were approved by the Institutional Animal Care and Use Committee (IACUC) at Texas A&M Health Science Center College of Medicine at Scott & White and conformed to the requirements of the Animal Welfare Act. Female 6–8-week-old NOD/*scid* obtained from Jackson Laboratory (Bangor, ME, USA) were used for this study. The mice were kept on a 12-h light–dark cycle.

### Cell culture

Human breast cell line HCC1806 (ATCC, Manassas, VA, USA; CRL2335) were cultured in high-glucose (4500 mg/ml) Dulbecco’s modified Eagle medium (PAA Laboratories GmbH, Pasching, Austria) supplemented with 10% fetal bovine serum (FBS, Biochrom AG, Berlin, Germany), gentamicine 10 *μ*g/ml and 2 mM glutamine as recommended by the supplier. The cells were then expanded by plating at 500 cells/cm^2^ in complete culture medium for MSCs except that FBS was reduced to 10%.^19,24^

iPS-MSCs were prepared as described previously.^9^ P3 iPS-MSCs were expanded in culture plasticware to obtain a P4 Master Bank. Cells from the P4 Master Bank were expanded through a further passage in culture plasticware or a final passage in a perfusion bioreactor (Quantum; Terumo), to generate a P6 Working Bank. Further expansion was on plasticware.

For each expansion, viable cells were recovered from frozen vials of about one million cells by thawing the vials and incubating them overnight in 15 cm diameter dishes and in Complete Culture Medium for MSCs (CCM). The CCM^19^ contained alpha-MEM (Gibco-BRL, Rockville, MD, USA), 17% FBS (lot selected for rapid growth; Atlanta Biologicals, Norcross, GA, USA), 1% penicillin and 100 *μ*g/ml streptomycin and supplemented with either 2 mM L-glutamine or GlutaMAX (Gibco). The cultures were washed with PBS, and adherent cells were lifted with 0.25% trypsin and 1 mM EDTA at 37 °C for 1 to 3 min. For expansion on plasticware, the cells were plated at about 500 cells/cm^2^ in CCM. The medium was replaced after 3 days, and the cells were harvested when the cultures were 70–90% confluent in 4–5 days. During the passages, the fold expansion varied from 16.3 to 29 and the population doublings from 4.0 to 4.9. Expansion in automated, closed and single-use Quantum Cell Expansion System bioreactor provided by Terumo BCT was performed in accordance with Termumo BCT standard protocols unless otherwise stated. After coating the inner circulatory system with tissue culture grade fibronectin (Sigma), 15×10^6^ cells were loaded onto the inner chamber of the bioreactor to achieve a theoretical density of 500 cells/cm^2^. The cells were allowed to attach for 24 h with inversion of the hollow fiber bioreactor and cultured in CCM containing a stabilized glutamine supplement (Glutamax, Thermo Fisher, Waltham, MA, USA), at an initial media replenishment rate of 0.1 ml/min. During the culture period, media replenishment was gradually raised to 0.8 ml/min to prevent lactate concentrations reaching 8 mmol/l. The cells were harvested on day 5 by 15 min of exposure to a commercially available trypsin replacement (TrypLE, Thermo Fisher). The fold expansion was 4.8 and the population doubling was 2.7.

### Vector design

Lentiviral constructs (System Biosciences, Mountain View, CA, USA) were based on a third-generation lentiviral vector modified with an insert that contained the CMV promoter driving the expression of CD/UPRT (17; InvivoGen, San Diego, CA, USA) combined with copGFP by P2A sequence.

### Lentivirus production, precipitation and titration

The lentiviral vectors were generated in HEK293T cells after transient transfection with the X-tremeGENE HP DNA Transfection reagent (Roche, Mannheim, Germany, Cat. No. 06366244001) and lentivector CD, pPACKH1 packaging mix plasmids (SBI, Mountain View, CA, USA, Cat. No. LV500A-1). Lentivirus-containing medium was collected after transfection for 2 days, and cellular debris was cleared by low-speed centrifugation at 1500×*g* for 5 min. The collected medium was concentrated with the PEG-it virus precipitation solution (SBI, catalog number LV810A-1), and pellets containing viral particles were resuspended with PBS. Lentiviral titers were measured with the Lenti-X qRT-PCR Titration Kit (Clontech, Mountain View, CA, USA, catalog number 631235).

### Transfection of iPS-MSCs

The iPS-MSCs in fresh culture medium were seeded 1×10^5^ cells per well in triplicate in six-well plates. Prior to transfection, the cells were incubated for 24 h at 37 °C in a humidified incubator in an atmosphere of 5–7% CO_2_. The culture medium was replaced with 1 ml fresh medium containing 100 *μ*g/ml of sterile-filtered protamine sulfate (catalog no. P4020-1G; Sigma-Aldrich, St Louis, MO, USA). The cells were infected with multiplicity of infection (MOI) 10, for 24 h, at 37 °C, and this step was repeated with an MOI of 10 for a further 24 h to achieve maximum transfection efficiency.^39^ After 48 h and two rounds of transduction, the cells were trypsinized and plated at 500 cells/cm^2^ in T175 cm^2^ flasks in MSC culture medium. The fresh culture medium was changed three times a week. After expansion to about 70% confluency in about 5 days, the cells were lifted with trypsin/EDTA and expanded further under the same conditions.

### cDNA synthesis and real-time quantitative PCR

The mRNA (1 *μ*g) was converted to cDNA using the High Capacity cDNA Reverse Transcription Kit (Applied Biosystems, Foster city, CA, USA). The expression of specific mRNAs was assayed using fluorescence-based real-time quantitative PCR (qPCR). qPCR reactions were performed using Fast or SYBR Green PCR Master Mix (Applied Biosystems) in triplicates for each sample. GAPDH was chosen as the reference gene. The amplification cycles were 95 °C for 5 s, 60 °C for 20 s and 72 °C for 15 s for copGFP and CD::UPRT, while the amplification cycles were 95 °C for 20 s, 95 °C for 2 s and 60 °C for 20 s for GAPDH and 18s. Primer probe numbers were catalog number Hs00266705_g1 for human GAPDH (Life Technologies); catalog number 4333760F for 18s eukaryotic endogenous control labeled with FAM probe (Applied Biosystems). Self-designed primers were used for the detection of copGFP with forward primer sequence 5′- CAGCGTGATCTTCACCGACAAG-3′ and reverse primer sequence 5′- GTCCACCACGAAGCTGTAGTAG-3′, with amplicon size 142 bp and for the detection of CD::UPRT gene, the forward primer sequence was 5′- CCTGGGACTCTACACCATCATC-3′ and reverse primer sequence was 5′- GTCAGTCTCCACAATCTGCTTCTG-3′, with amplicon size 139 bp. The SYBR Green master mix (Applied Biosystems) was used for the amplification of copGFP and CD::UPRT gene. At the end of the assay, a melting curve was constructed to evaluate the specificity of the reaction. All quantitative real-time PCR reactions were performed and analyzed using the StepOne Plus Real-Time PCR System (Applied Biosystems) with the comparative ^∆∆^Ct method.

### Cells labeling

In the co-culture experiments, the iPS-MSCs-CD cells were labeled with copGFP while HCC1806 cells were labeled with CellTracker Red (catalog number C34552, Thermo Fisher Scientific, Waltham, MA, USA) as recommended by the manufacturer. Very briefly, cells were washed with PBS and 10 *μ*M dye was added to one million cells for 30 min. Afterwards, the unattached dye was removed by PBS washing and centrifugation. For the detection of dead cells, propidium iodide dye (catalog number P3566, Thermo Fisher Scientific) or trypan blue dye (catalog number 15250061, Thermo Fisher Scientific) was used according to the manufacturer’s instructions.

### Histology

Mice were anesthetized with 3% isoflurane in 100% oxygen until anesthetized and killed by i.p. injections of a solution of 80 mg/kg of ketamine and 8 mg/kg of xylazine. Formalin-fixed, paraffin-embedded lung tissues were cut into 8 *μ*m thick sections, stained with hematoxylin/eosin and evaluated by light microscopy.

### Western blotting

Protein isolation was performed by cell lysis using RIPA buffer (Sigma-Aldrich) in the presence of protease inhibitors (Roche). Proteins were resolved by electrophoresis on 12% sodium dodecyl sulfate-polyacrylamide gels and transferred onto polyvinylidene fluoride membranes (Hybond-P, Thermo Fisher Scientific). After blocking in PBS containing 0.1% Tween 20 (PBS-T) supplemented with 3% bovine serum albumin for 1 h, membranes were incubated with the following primary antibodies at 4 °C overnight: anti-β actin (catalog number A1978; Sigma-Aldrich), anti-TurboGFP (catalog number PA5-22685; Life Technologies) and CD (catalog number. CD PA1-85365; Thermo Fisher Scientific). Subsequently, membranes were rinsed with PBS-T and incubated with the species-specific horseradish peroxidase-coupled secondary antibodies (dilution 1 : 10000; Jackson ImmunoResearch Laboratories, West Grove, PA, USA) for 1 h at room temperature. Blots were developed using enhanced chemoluminescence reagents (SuperSignal, Thermo Scientific) and the ChemiDoc XRS+ system (Bio-Rad, Hercules, CA, USA).

### Tumor initiating and growth

In one experiment, the HCC1806 breast cancer cells (1×10^6^) and iPS-MSCs (1×10^6^) were co-injected into the fourth mammary fat pad of 6–8-week-old NOD/*scid* mice, followed by i.p. injection of 3.0 or 1.5 mg of 5-FC in 1.0 ml PBS for 5 consecutive days (treated group). The control group received i.p. injections of PBS. The tumor size was determined every 3 days with a vernier caliper. After 28 days, the mice were killed and the tumors were excised and weighed. Tumor volume was calculated by using the formula: tumor volume (mm^3^)=1/2 length (mm)×width (mm)^2^.^40^ In another set of experiments, 1×10^6^ HCC1806 cells were injected into the mammary fat pad of 6–8-week-old NOD/*scid* mice to develop tumors first. After 10 days, 1×10^6^ iPS-MSCs transduced with the CD gene were injected into the tumors or adjacent tissue. On the same day, 5-FC injections were started and continued for 5 consecutive days. After 26 days, the tumors were harvested, weighed and size measured as above.

### Statistics

Student’s *t*-test was used to compare two groups. One-way ANOVA was used to compare several groups using GraphPad Prism 5 (GraphPad Software, San Diego, CA, USA). Comparison of tumor frequency between the experimental and control groups was analyzed by a two-tailed Fisher’s Exact test. All *in vitro* experiments were carried out in triplicate unless specified. *P*<0.05 was considered statistically significant.

## Notes

DJP is chair of the scientific advisory committee and has a small equity position in a biotech company (Temple Therapeutics LLC) with an interest in MSCs. The remaining authors declare no conflict interest.
