# iPS-derived MSCs from an expandable bank to deliver a prodrug-converting enzyme that limits growth and metastases of human breast cancers

**DOI:** 10.1038/cddiscovery.2017.29

**Published:** 2017-08-21

**Authors:** M Ullah, Y Kuroda, T J Bartosh, F Liu, Q Zhao, C Gregory, R Reger, J Xu, R H Lee, D J Prockop

**Correction to:** *Cell Death Discovery* (2017) **3**, 16064; doi:10.1038/cddiscovery.2016.64; published online 6 February 2017

Since the publication of this article, it has been noted that the following acknowledgement was missing:

Mujib Ullah and Yasumasa Kuroda contributed equally to the work.

The authors apologise for this error.

The original html and pdf versions of the paper have been rectified, and now carry the corrected paper.

